# Tumor Invasion of *Salmonella enterica* Serovar Typhimurium Is Accompanied by Strong Hemorrhage Promoted by TNF-α

**DOI:** 10.1371/journal.pone.0006692

**Published:** 2009-08-20

**Authors:** Sara Leschner, Kathrin Westphal, Nicole Dietrich, Nuno Viegas, Jadwiga Jablonska, Marcin Lyszkiewicz, Stefan Lienenklaus, Werner Falk, Nelson Gekara, Holger Loessner, Siegfried Weiss

**Affiliations:** 1 Molecular Immunology, HZI – Helmholtz Centre for Infection Research, Braunschweig, Germany; 2 Department of Internal Medicine I, University of Regensburg, Regensburg, Germany; University of Toronto, Canada

## Abstract

**Background:**

Several facultative anaerobic bacteria with potential therapeutic abilities are known to preferentially colonize solid tumors after systemic administration. How they efficiently find and invade the tumors is still unclear. However, this is an important issue to be clarified when bacteria should be tailored for application in cancer therapy.

**Methodology/Principal Findings:**

We describe the initial events of colonization of an ectopic transplantable tumor by *Salmonella enterica* serovar Typhimurium. Initially, after intravenous administration, bacteria were found in blood, spleen, and liver. Low numbers were also detected in tumors associated with blood vessels as could be observed by immunohistochemistry. A rapid increase of TNF-α in blood was observed at that time, in addition to other pro-inflammatory cytokines. This induced a tremendous influx of blood into the tumors by vascular disruption that could be visualized in H&E stainings and quantified by hemoglobin measurements of tumor homogenate. Most likely, together with the blood, bacteria were flushed into the tumor. In addition, blood influx was followed by necrosis formation, bacterial growth, and infiltration of neutrophilic granulocytes. Depletion of TNF-α retarded blood influx and delayed bacterial tumor-colonization.

**Conclusion:**

Our findings emphasize similarities between Gram-negative tumor-colonizing bacteria and tumor vascular disrupting agents and show the involvement of TNF-α in the initial phase of tumor-colonization by bacteria.

## Introduction

Bacteria and bacterial products have been investigated as therapeutic agents to treat solid tumors for more than 100 years [1]–[3]. In this context, several bacterial species have been discovered that selectively accumulate and replicate in solid tumors [4]–[8]. As reason was suggested that such bacteria find favorable niches within the hypoxic and necrotic regions of the tumor. Accordingly, tumor-colonizing bacteria are either obligate or facultative anaerobic.

Presently, tumor-colonizing bacteria have attracted sincere investigative interest due to their obvious therapeutic potential. The use of viable migratory bacteria that bear intrinsic or engineered tumor therapeutic effectors might circumvent several problems encountered with conventional tumor therapies. For instance, due to the irregularly developed vasculature the accessibility of some tumor regions for chemotherapeutics might be reduced. Thus, the tumor is not affected completely and is able to continue to grow. Similarly, hypoxic regions as often found in solid tumors, pose problems for ionizing irradiation therapy since oxygen is required for its effectiveness. Bacteria that thrive under anaerobic conditions would not be limited by this.

Alternatively, isolated microbe-associated molecular patterns (MAMPs) have been investigated to activate the immune system against the cancer are thus limited [9]–[11]. Some success has been observed. However, viable bacteria have several advantages compared to such “passive molecules” and are therefore able to colonize the tumor and actively destroy it at least in part. In addition, tumor colonization and therapeutic properties of bacteria can be improved either by selection or genetic engineering [4], [12], [13].

Various bacteria have been shown to be usable for bacteria-mediated tumor therapy [14]. For instance, spores of Gram-positive Clostridia spp. that exclusively germinate in necrotic parts of tumors have been employed alone or in combination with radio- and chemotherapy for treatment of experimental tumors [15]–[19]. Similarly, Gram-negative *Salmonella enterica* serovar Typhimurium (*S.* Typhimurium) as well as *Escherichia coli* (*E. coli*) strains have been studied. Thus, a highly attenuated *S.* Typhimurium strain VNP20009 was established and tested in animal models. Importantly, this strain was found to be safe in first clinical trials [20], [21]. However, the outcome of such trials was rather disillusioning despite the success in experimental models [22]–[24]. Only very high doses or continuous administration of VNP20009 led to tumor colonization. Therapeutic effects were rare [20], [21]. Nevertheless, these first clinical trials showed the general applicability of bacteria in cancer therapy. However, they also made clear that it is essential to understand more about the interactions between tumor-bearing host and bacteria in order to render bacteria safe and efficient.

Presently, the major drawback regarding bacterial tumor therapy is the insufficient knowledge about bacterial entry into the tumor. Consistently, *Salmonella* were shown to adhere to walls of blood vessels at low flow rates [25] and to be attracted to necrotic areas of *in vitro* tumors [26]. This suggested the production of chemotactic compounds by quiescent or dead cancer cells inside solid tumors.

Once in the tumor, *E. coli* for instance altered the tumor microenvironment in murine breast tumors by vascular remodeling, focal concentration of tumor associated macrophages and focal expression of MMP-9 and TNF-α around bacterial colonies [27].

In our own studies, we observed a massive infiltration of CD11b^+^Gr1^+^ host neutrophilic granulocytes into ectopic colon carcinomas after infection with different Gram-negative facultative anaerobic bacteria [28]. Such host cells formed a barrier restricting bacteria to necrotic areas and prohibited bacterial spread within the tumor. Importantly, bacterial colonization clearly led to a severe enhancement of necrosis [28].

In the present study we investigated the initial events of tumor colonization by *S.* Typhimurium SL7207 to obtain insights into the mechanism of tumor entry. As early as 30 min post infection (p.i.) first bacteria were observed in tumor tissue. In parallel high concentrations of TNF-α could be found in blood and a tremendous influx of blood into the tumor was observed at that time. This was followed by necrosis formation, bacterial growth, and neutrophil-infiltration into the tumor. Our observations indicate that tumor-colonizing *Salmonellae* provoke effects in tumor blood vessels that resemble vascular disrupting agents (VDAs) or TNF-α. Our data further suggest that TNF-α in concert with other pro-inflammatory cytokines plays an important role in efficient bacterial tumor colonization. As this study presents essential first findings on how Salmonella enter tumors, it represents an important basis for further investigations required to render Salmonella effective and safe for clinical application in cancer therapy.

## Materials and Methods

### Ethics statement

All animal experiments were performed according to local guidelines from LAVES (Niedersaechsisches Landesamt für Verbraucherschutz und Lebensmittelsicherheit).

### Bacterial strains and growth conditions

The *S.* Typhimurium strain SL7207 (hisG, ΔaroA) was kindly provided by Bruce Stocker [29] and *E. coli* TOP10 was purchased from Invitrogen. Both bacterial strains were grown in LB medium.

### Cell lines and animals

BALB/c mice were purchased from Harlan (Borchem). CT26 colon carcinoma cells (ATCC CRL-2638) were grown as monolayers in IMDM Medium (Gibco BRL, Germany) supplemented with 10% (v/v) heat-inactivated fetal calf serum (Integro), 250 µmol/l β-Mercaptoethanol (Serva) and 1% (v/v) penicillin/streptomycin (Sigma-Aldrich, Germany).

### Infection of tumor bearing mice and recovery of bacteria from tissues and blood

Six week old, female BALB/c mice were subcutaneously inoculated at the abdomen with 5×10^5^ CT26 cells. Mice bearing tumors of approximately 4–7 mm diameter were intravenously injected with 5×10^6^ CFU of *S*. Typhimurium suspended in phosphate-buffered saline (PBS). At different time points p.i. mice were sacrificed and their tumors and spleens were transferred into 1 ml of sterile ice-cold PBS containing 0.1% (v/v) Triton X-100, livers were transferred into 2 ml of this solution. Tissues were homogenized by using a Polytron PT3000 homogenizer (Kinematica). In addition, heart blood was taken after sacrifice and diluted 1∶2 with 0.1% (v/v) Triton X-100. For determination of total CFU per organ, homogenates were serially diluted in PBS and plated with the required antibiotics on LB plates. Tumors were additionally weighed to calculate the total CFU per gram tumor tissue.

### Non-invasive in vivo imaging of luminescent Salmonella

An *S.* Typhimurium strain expressing the luxCDABE operon of *Photorhabdus luminescens* under the control of the β-Lactamase promoter was used to visualize the bacterial dissemination in the living animal with the help of the IVIS200 system (Calipers). Prior to analysis, mice were anaesthetized with 2% isoflurane using XGI-8 gas anesthesia system (Calipers). Pseudocolored images of photon counts and photographic images were obtained according to instructions of the manufacturer. The software Living Image 2.5 (Calipers) was used for image analysis.

### Determination of Hemoglobin content in tumors

Tumors were removed and homogenized in 1 ml ice-cold sterile PBS. To lyse erythrocytes 500 µl ACK lysing Buffer were added to the homogenate and incubated for 10 minutes at room temperature. Subsequently, the samples were centrifuged at 13,000 rpm for 10 minutes and 300 µl of the supernatants were mixed with 1 ml Drabkin's reagent (Sigma-Aldrich). After 30 min of incubation the optical density at 540 nm was measured.

### Determination of TNF-α in serum

TNF-α biological activity in serum was determined using TNF sensitive L929 fibroblasts. In brief: 4×10^4^ cells were seeded in 96-well plates and incubated over night. The following day the medium was replaced with 100 µl complete IMDM including the samples of interest and actinomycin D [6.25 µg/ml] (Sigma-Aldrich). 50 µl of TNF-α standard [1 ng/ml] (CHEMICON) as a reference and 15 µl of serum samples were added in duplicates to the cells and serial dilutions were performed. After 18 hours of incubation, cell supernatants were replaced with 100 µl complete IMDM and cell viability was determined using the EZ4U KIT (BIOMEDICA). TNF-α concentrations were defined by comparing the L929 killing induced by the serum samples, with the killing induced by dilutions of TNF-α of known concentrations.

### RT-PCR of Salmonella-infected CT26 cells and subcutaneous CT26-tumors

For *in vitro* experiments, CT26 cells were infected with *S*. Typhimurium SL7207 for 30 min before changing antibiotic-free IMDM medium to IMDM medium containing 50 µg/ml gentamycin. At different time points after infection cells were lysed and RNA was isolated using RNAeasy Mini Kit according to the manufacturer's protocol (Qiagen). Reverse transcription was carried out using Revert Aid First Strand cDNA Synthesis kit (MBI Fermentas). For *in vivo* experiments, BALB/c mice bearing subcutaneous CT26-tumors were intravenously infected with 5×10^6^ SL7207. At different time points after infection tumors were removed and homogenized in RNAprotect (Qiagen). After centrifuging the homogenate for 5 min at 13 000 rpm the supernatant was used to isolate RNA and reverse transcription was performed as described above. Semi-quantitative PCRs were performed using primers; 5′*-*TCT CAT CAG TTC TAT GGC CC-3′, 5′ *-GGG AGT AGA CAA GGT ACA-3.′* for TNF-α (amplifying a fragment of 212 bp) and 5′*-*CTG GAC GAG GGC AAG ATG AAG C-3′, 5′ *-TGA CGT TGG CGG ATG AGC ACA-3.′* for ribosomal protein S9 (RPS9). Amplification conditions were: denaturation at 94°C for 1 min followed by 27 and 32 cycles (for RPS9 and TNF-α), respectively, of repeated denaturation (20 s at 94°C), annealing (20 s at 58°C) and extension (20 s at 72°C). PCR products were analyzed on a 2% agarose gel. Equal amounts of cDNA were applied according to intensity of the amplification product of RPS9.

### Determination of proinflammatory cytokines and chemokines in serum

Cytokines were quantified using a cytometric bead array (CBA) mouse inflammation kit from BD Bioscience. For the assay, 50 µl of PE detection reagent were added to 50 µl of mixed solution of IL-6, IL-10, MCP-1, IFN-γ, TNF-α and IL-12(p70) detection beads. A volume of 50 µl of each sample was transferred to the appropriate assay tubes containing mixed capture beads and PE detection reagent. The assay tubes were incubated protected from light for 2 h at RT. A volume of 1 ml of wash buffer was added and the tubes were centrifuged at 200×g for 5 minutes. The supernatant was discarded and the samples were resuspended in 300 µl of wash buffer. The acquisition was performed in a FACSCalibur (BD Bioscience) using the CBA software. Standard curves were plotted in each experiment for all of the analyzed cytokines.

### TNF-α studies

Six week old, female BALB/c mice were subcutaneously inoculated at the abdomen with 5×10^5^ cells of the colon adenocarcinoma cell line CT26. Ten days after injection of tumor cells one group of mice was treated i.v. with graded amounts of recombinant TNF-α (less than 0.1 ng endotoxin per mg TNF-α; Chemicon) and the second group received graded amounts of TNF-α directly applied into the tumor (i.t.). Anti-TNF-α treatment was carried out as follows: tumor-bearing mice were injected i.v. with either 250 µg rat anti-TNF-α (clone TN3-19.12, BioLegend) or 200 µg of rat anti-mouse TNF-α (Clone V1qH8 [30]) or normal rat IgG as control 20 min before an i.v. injection of 5×10^6^ CFU of *S*. Typhimurium. Tumors were photographed at different time points p.i., analyzed histologically, used for CFU determination, and for determination of hemoglobin content, respectively.

### Histology

Tumors were removed from sacrificed mice and snap-frozen in Tissue-Tek OCT Compound (Sakura Finetek). Cryosections of 10 µm were cut with a microtome-cryostat and placed onto glass slides. Slides were air dried at room temperature overnight and fixed in acetone at −20°C for 3 min. Slides were rehydrated in PBS, blocked with 50 µg/ml BSA and 1 µg/ml FcR blocker (rat anti-mouse CD16/CD32), and stained with the following reagents: polyclonal rabbit anti-*S.* Typhimurium (USBiological), polyclonal goat anti-rabbit Alexa 488 (Sigma-Aldrich), rat anti-Gr1 biotin (RB6-8C5 – Ly6G-specific, eBioscience), Streptavidin-Cy5 (Invitrogen), rat anti-CD31 PE (eBioscience). After staining, the slides were washed, dried, mounted with mounting medium (Neomount, Merck) and analyzed using a laser scanning confocal microscope (LSM 510 META, Zeiss). Images were processed with LSM5 Image Browser (Zeiss) and Adobe Photoshop 7.0. For paraffin sections, tumors were fixed in 10% (v/v) paraformaldehyde and embedded in paraffin wax. 5 µm sections were mounted on slides and stained with hematoxilin and eosin (HE). The stained paraffin sections were analyzed with an Olympus BX51 microscope and pictures were taken with an Olympus U-CMAD3 camera.

### Statistical analysis

Statistical analysis were performed using the two-tailed Student's t-test with P<0,05 considered as significant.

## Results

### Tumor colonization of *S.* Typhimurium SL7207 within the first 24 h post infection

**Figure 1:**
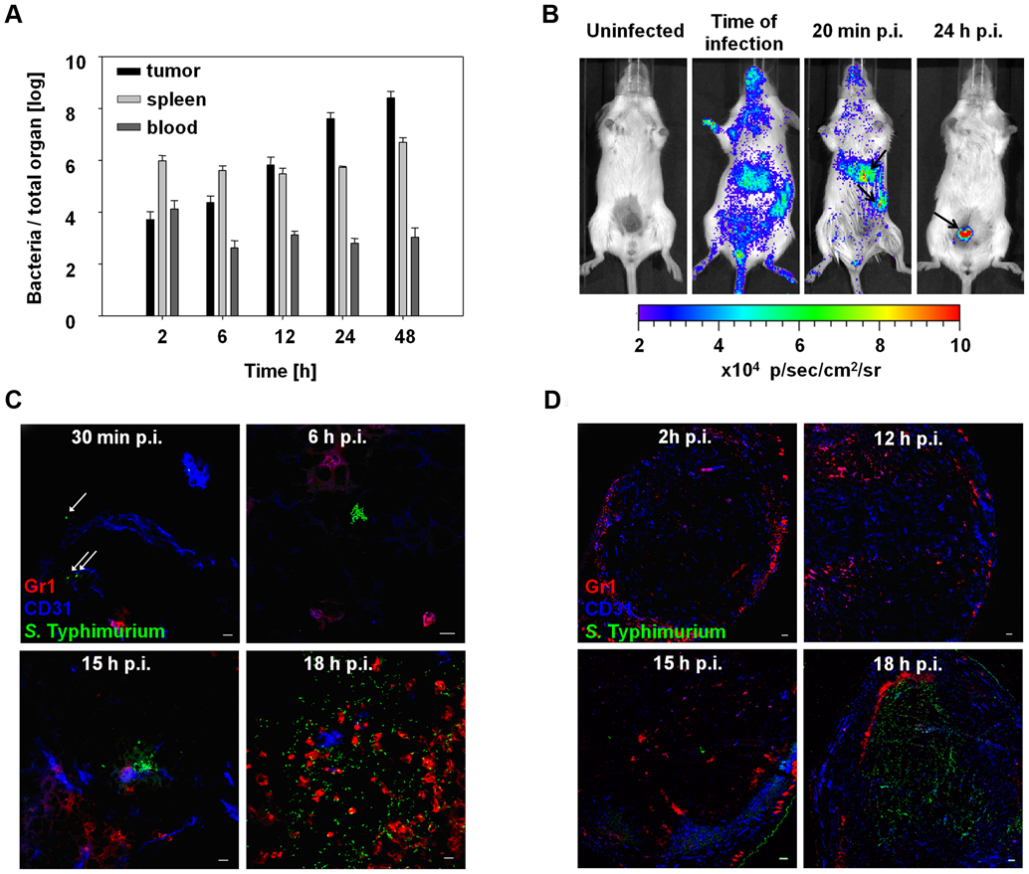
Time course of bacterial accumulation in different organs and bacterial colonization of solid CT26 tumors. Tumor bearing mice were infected i.v. with *S.* Typhimurium SL7207. (a) 2 h, 6 h, 12 h, 24 h and 48 h p.i. tumor and spleen were homogenized. Tumor, spleen and blood were plated and the CFUs per total organ were determined. (b) Non-invasive *in vivo* imaging of bacterial bioluminescence. Tumor-bearing mice were infected with *S.* Typhimurium expressing the luxCDABE operon of *Photorhabdus luminescens* under the control of the β-Lactamase promoter. Arrows show sites of high bacterial accumulation. Images were taken at the indicated time points. (c) High magnification images of tumor cryosections at the indicated time points p.i. with *S.* Typhimurium SL7207. In all pictures, bacteria are stained in green, blood vessel endothelial cells are stained in blue and neutrophilic granulocytes are stained in red. White arrows point at individual Salmonella. White bars correspond to 10 µm in all pictures. (d) Overviews of CT26 tumor cryosections at the indicated time points p.i. with *S.* Typhimurium SL7207. Stainings are described in (c). White bars correspond to 100 µm in all pictures. Experiments were repeated at least three times with identical results.

Although it is well known that several bacterial strains are able to colonize solid tumors and exert potential anti-tumoricidal effects, the mechanism by which bacteria enter tumors is not clear so far. As this knowledge is essential to render bacteria safe and efficient for anti-cancer therapy we investigated the early time points after systemic Salmonella administration in a murine tumor model. To this end, BALB/c mice bearing the commonly used murine colon carcinoma CT26 were infected i.v. (intravenously) with 5×10^6^ *S.* Typhimurium SL7207 a strain that is widely used as carrier for vaccine experiments. At different time points p.i. (post infection), colony forming units (CFU) in blood, tumor, and spleen were determined (Fig. 1a). In blood, bacterial numbers quickly decreased within the first 6 h p.i. but then remained constant until the end of the observation period. In spleen, the number of *Salmonellae* reached a plateau already at 2 h p.i. and showed only minor variations over the observation period. In tumors, bacterial numbers increased constantly from 2 h to 48 h p.i. most likely due to proliferation.

This course of colonization was corroborated by non-invasive *in vivo* imaging using *S.* Typhimurium equipped with the lux operon of *Photorhabdus luminescens*. Bio-luminescence distributed over the entire body could be observed immediately after i.v. administration. However, already 20 min p.i. significantly increased luminescence was detectable in spleen and liver indicating colonization (Fig. 1b arrows). Colonization of the subcutaneous tumors located at the lower abdomen of the mice became apparent later. By 24 h p.i. the signal obtained from tumors (arrow) was by far exceeding the signal from spleen and liver as bacteria were accumulating in high numbers at the tumor site (Fig. 1b).

To obtain a more detailed insight into initiation of tumor colonization, we refrained to histological analysis. Cryosections of tumors from various time points were stained with fluorescence-conjugated antibodies against *Salmonellae*, neutrophilic granulocytes (CD11b^+^Gr1^+^) and CD31, a molecule found on endothelial cells to highlight distribution and course of blood vessels. Single bacteria could be found inside blood vessels of CT26 tumors 30 min p.i. (Fig. 1c, 30 min). Interestingly, some bacteria had already escaped from such vessels. This did not change significantly over the first 6 h p.i.. Most of bacteria were single and located inside or in close contact to blood vessels. Rarely, small clusters or colonies of *Salmonellae* were detected (Fig. 1c 6 h p.i.). Such clusters were observable more frequently at 12 h p.i. (data not shown) and they were distributed over the entire tumor although mostly the bacterial colonies were still in close contact to blood vessels. By 15 h p.i. the scattered bacterial colonies had become considerably larger (Fig. 1c, 15 h p.i.) and single bacteria began to localize distantly from blood vessels. By 18 h p.i. this “initial phase” concluded (Fig. 1c, 18 h p.i.). The distribution of bacteria and immigrated Gr1^+^ host neutrophils now resembled the situation that was described for 48 h and later [28].

Low magnification overviews of colonized CT26 tumors revealed immigration and distribution of host neutrophils (Fig. 1d). At early time points p.i. Gr1^+^ cells were distributed in low numbers over the entire tumor with a slight accumulation at the tumor rim. The formation of bacterial colonies and the dissemination of bacteria within the tumor correlated with a strong influx of neutrophils between 12 h and 15 h p.i.. By 18 h p.i. a small barrier of neutrophils had formed that apparently shielded the vital tumor tissue from the now quickly multiplying *Salmonellae.* The basic pattern of a neutrophil barrier surrounding the bacteria-colonized necrosis, which we had reported for two days p.i. [28], could already be seen after 18 h p.i., albeit less pronounced.

Taken together: After i.v. application of *S*. Typhimurium into tumor bearing mice bacteria very quickly accumulate in spleen and liver while solid tumors are colonized only by limited numbers at that time. During the course of infection bacterial numbers in the normal target organs remain rather constant. In contrast, numbers in the tumor dramatically increase during this period most likely due to proliferation. This is accompanied by a strong influx of host neutrophils.

### Bacterial colonization of solid tumors is accompanied by hemorrhage

**Figure 2:**
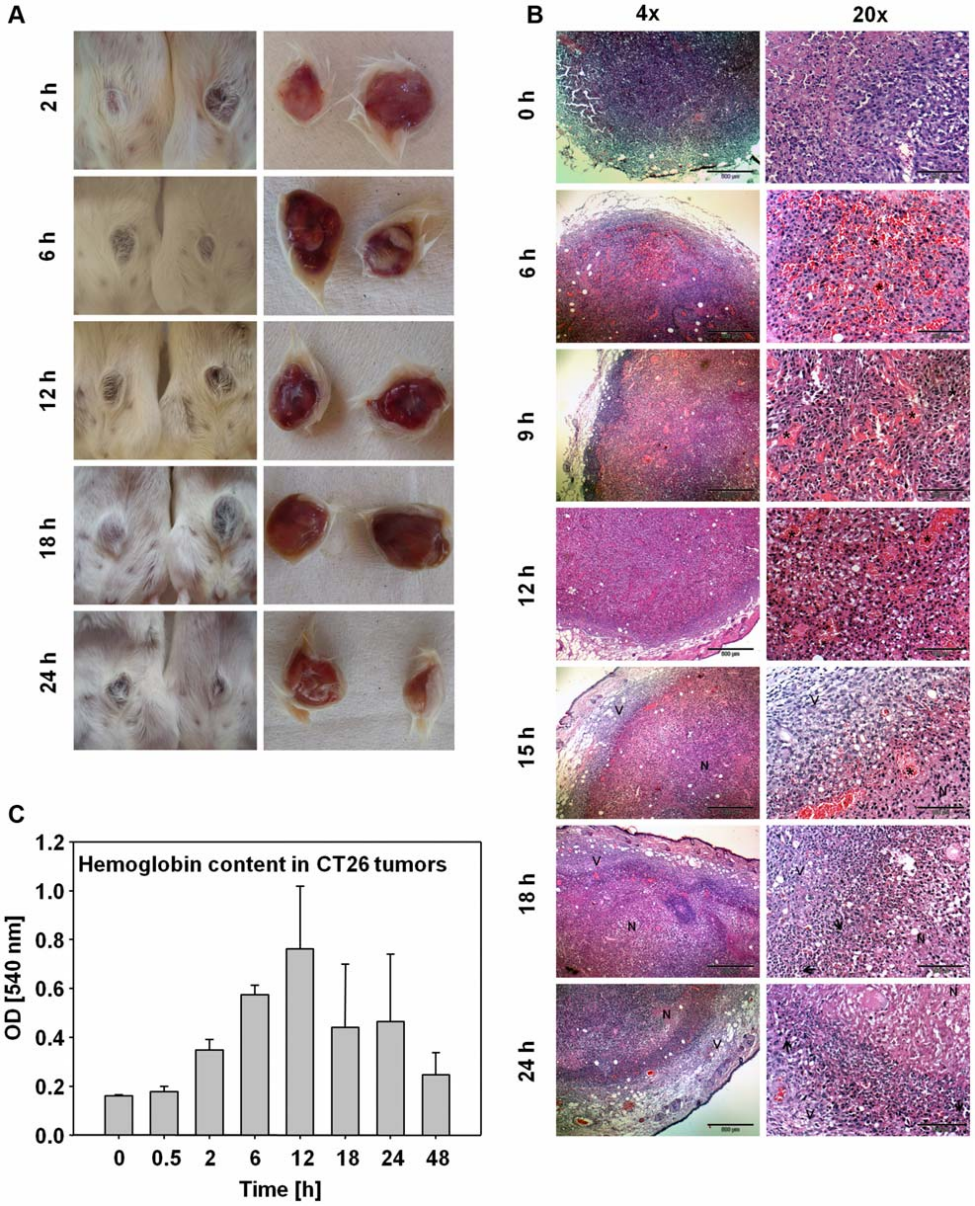
Time course of blood influx into CT26 tumors after bacterial infection. (a) Photographs of CT26 tumors at different time points p.i. with *S.* Typhimurium. (a left) Photographs of the fur side of the tumors. (a right) Photographs of the ventral side of the tumors. (b) HE-stained paraffin sections of CT26 tumors at different time points p.i. with *S.* Typhimurium. (b left) Low magnification overviews. (b right) Higher magnifications of the tumors shown in (b left). 6 h–12 h: High magnifications of erythrocyte-containing (stars) central tumor areas. 15 h–24 h: High magnifications of the transition between developing necrosis (N) and remaining vital tumor rim (V). Arrows point exemplarily at neutrophils. Black bars correspond to 500 µm in (b left) and 100 µm in (b right). (c) Hemoglobin content in CT26 tumors at different time points p.i. with *S.* Typhimurium. Bars show standard deviations of means. Experiments were repeated three times with identical results.

A first hint on the mechanism of tumor colonization by *S.* Typhimurium came from macroscopic observations. Between 6 h and 15 h p.i., tumors became dark red indicating strong blood influx (Fig. 2a), which started to resolve at about 24 h p.i.. To investigate this phenomenon in more detail, we analyzed HE-stained paraffin sections (Fig. 2b) and found high numbers of erythrocytes (indicated by stars) at various places of the tumor at 6 h p.i.. Between 9 h and 15 h p.i. erythrocytes inside the tumor disintegrated. The complete tumor center that contained disintegrating erythrocytes became necrotic (indicated by “N”) as can be seen by the purple staining in Fig. 2B (15 h–24 h). The outer rim of the tumors remained vital (indicated by “V”). In accordance with the cryosections (Fig. 1c and d), neutrophils infiltrated the tumors between 18 h and 24 h p.i. and settled at the transition between viable and necrotic tumor regions (arrows point at exemplary neutrophils).

To independently confirm these observations, the hemoglobin content in CT26 tumors after infection with *Salmonellae* was determined (Fig. 2c). This should be directly correlated with influx of blood. Strong increases of hemoglobin between 2 h and 12 h p.i. were observed confirming an accumulation of blood in the tumors. After 12 h p.i. the hemoglobin content in the tumors decreased, pointing at disintegration and decomposition of the infiltrated erythrocytes.

These observations suggested that shortly after i.v. administration of *Salmonellae* to tumor-bearing mice a severe hemorrhage is induced inside the tumors that might flush in blood-borne bacteria. Upon resolution, a large necrotic area is left behind in which bacteria reside and thrive, and which is enclosed by host neutrophils.

### Induction of high concentrations of TNF-α upon administration of *S.* Typhimurium

**Figure 3:**
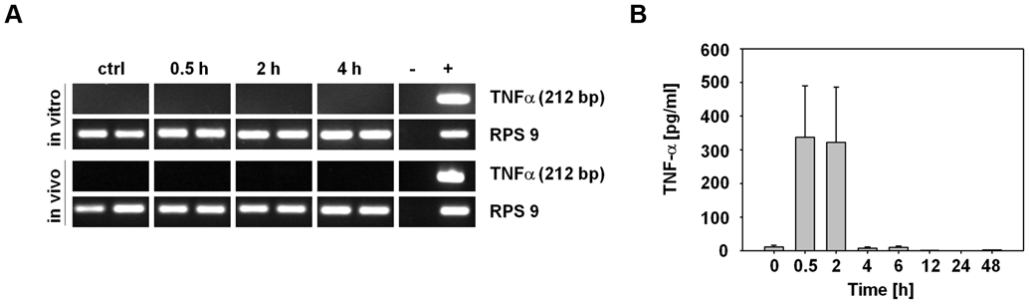
TNF-α release into the blood of *S.* Typhimurium-infected, CT26 tumor-bearing mice. (a) RT-PCRs were performed with RNA isolated from *S.* Typhimurium-infected CT26 cells (*in vitro*) and from CT26 tumors of *S.* Typhimurium-infected, tumor-bearing BALB/c mice (*in vivo*) at the indicated time points. The amplification product has a size of 212 bp. (b) TNF-α concentration in the blood of CT26 tumor-bearing BALB/c mice at different times p.i. determined using TNF sensitive L929 fibroblasts. Error bars show standard deviations of means. Results are representative for at least two independent experiments with 3–5 mice per group.

The strong hemorrhage of tumors and the subsequent infiltration of neutrophils suggested the induction of blood vessel-disrupting, neutrophil-attracting factors by the infected solid tumors. One cytokine that is known to promote such phenomena is TNF-α [31]. However, neither RT-PCRs from *Salmonella*-infected CT26 cells *in vitro*, nor RT-PCRs from *Salmonella*-infected CT26 tumors *in vivo* showed expression of TNF-α by CT26 cells or immune cells residing in CT26 tumors (Fig. 3a). Nevertheless, in blood we could detect TNF-α shortly after administration of bacteria (Fig. 3b). Here, TNF-α levels peaked between 30 min and 2 h p.i. and reached background levels again by 4 h p.i..

### Systemic administration of TNF-α leads to hemorrhage in tumors

**Figure 4:**
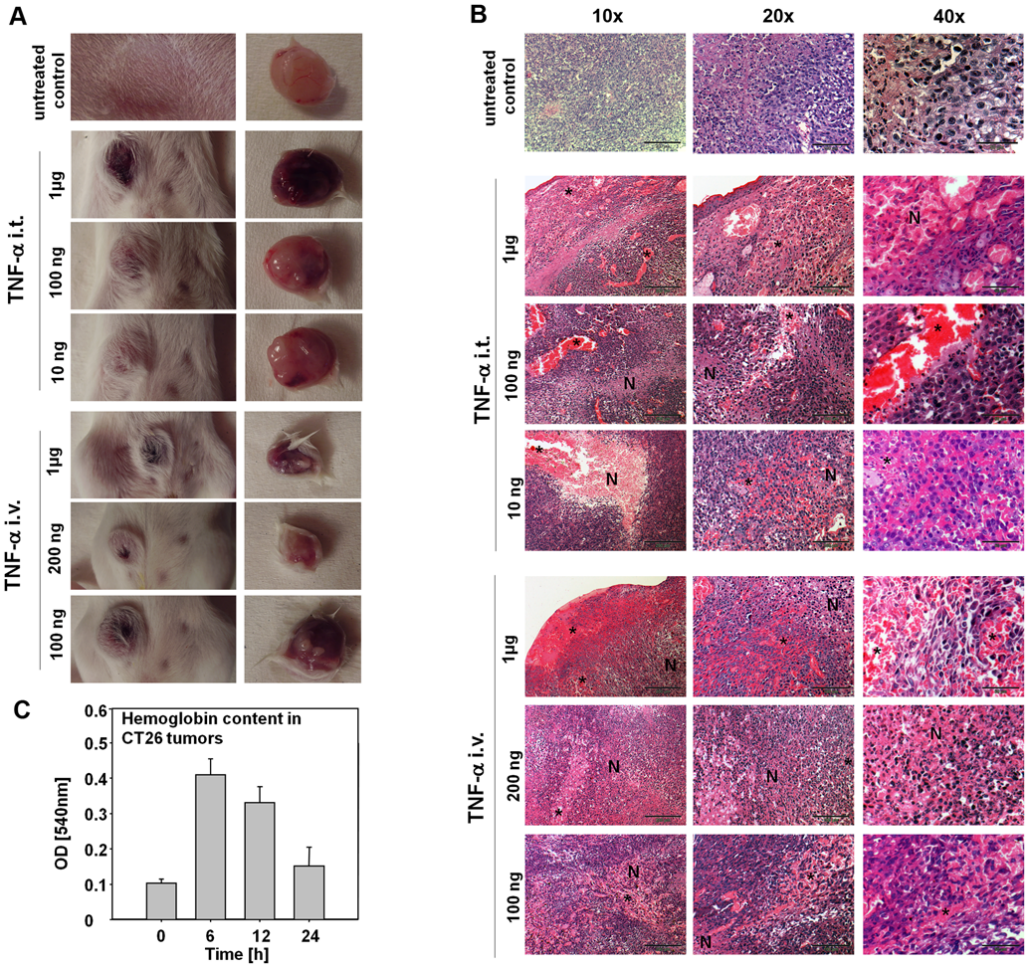
Blood influx into CT26 tumors after treatment with TNF-α. CT26 tumor-bearing BALB/c mice were treated i.v. or i.t. with the indicated amounts of recombinant TNF-α. Photographs were taken and histology was performed 6 h post treatment. (a) Photographs of tumors of differently treated mice. (a left) Photographs of the fur side of the tumors. (a right) Photographs of the internal side of the tumors. (b) HE-stained paraffin sections of the tumors shown in (a). The black bars correspond to 200 µm in the 10×magnification pictures, to 100 µm in the 20×magnification pictures and to 50 µm in the 40×magnification pictures. (N) stands for necrotic region, the stars show accumulations of erythrocytes.(c) Hemoglobin content in CT26 tumors of BALB/c mice after injection of 200 ng TNF-α i.v.. Results are representative for at least two independent experiments with 3–5 mice per group.

To test whether TNF-α in blood could be responsible for hemorrhage of CT26 tumors, graded concentrations of murine TNF-α were injected i.v. or i.t. (intratumorally) into tumor-bearing mice. Indeed, even the lowest amount of TNF-α injected, which was not detectable in the blood of the animals upon injection (data not shown), was sufficient to induce influx of blood into the tumors (Fig. 4a and 4b). HE-stained paraffin sections confirmed the presence of high numbers of erythrocytes (indicated by stars) inside tumors by 6 h post injection (Fig. 4b), similar to the observations made after administration of *S.* Typhimurium. Large amounts of erythrocytes could be observed in all samples from treated mice. Tumor tissue exposed to hemorrhage is apparently becoming necrotic (indicated by “N). Since the recombinant TNF-α was essentially free of endotoxin, hemorrhage was obviously induced exclusively by this cytokine.

Determination of hemoglobin content in CT26 tumors after i.v. injection of 200 µg TNF-α into tumor bearing mice confirmed these results (Fig. 4c). Actually, the kinetics of blood influx closely resembled that observed when bacteria were administered i.v.. A quick rise by 6 h to 12 h and resolution by 24 h post application was observed. Thus, TNF-α per se is able to induce a similar hemorrhage in CT26 tumors as i.v.-injected *Salmonellae*. Interestingly, the extend of blood influx induced by TNF-α was lower than the influx observed after bacterial infection. This might suggest the involvement of additional mechanisms induced by the microorganisms.

### Depletion of TNF-α retards blood influx and bacterial tumor colonization

**Figure 5:**
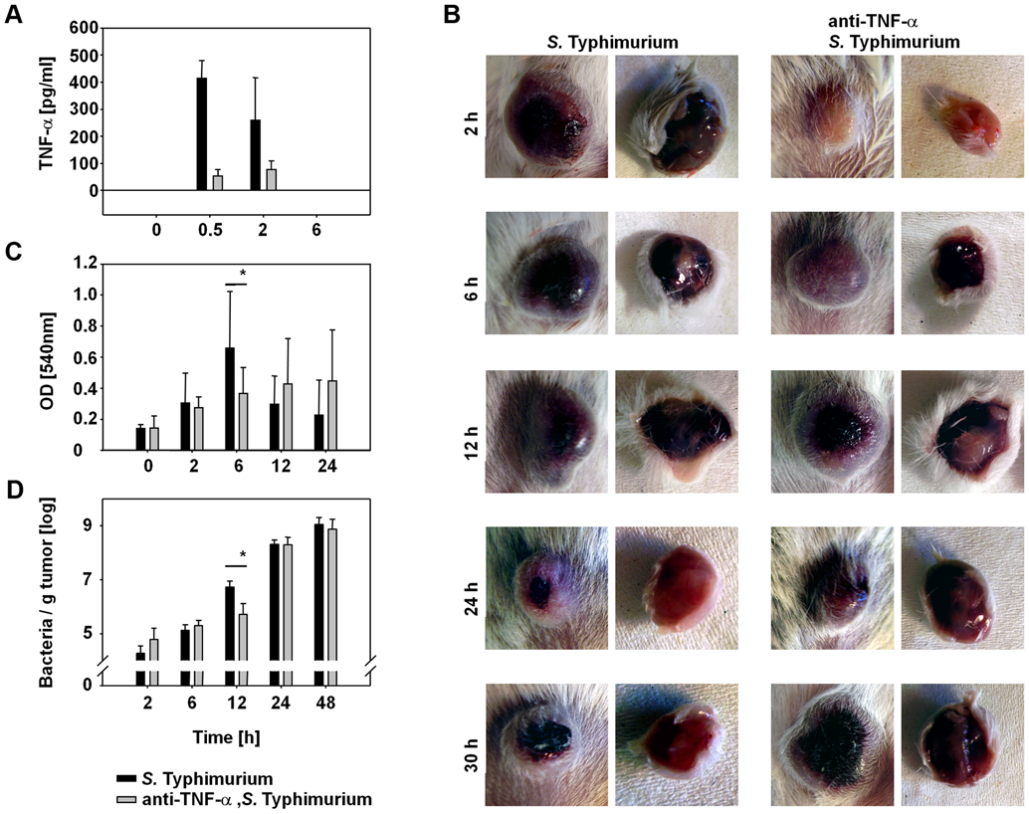
Inhibition of TNF-α in *S.* Typhimurium-infected BALB/c mice retards blood influx into tumors and bacterial colonization. (a) TNF-α concentration in the blood of *S.* Typhimurium-infected, CT26 tumor-bearing BALB/c mice (black bars) and of *S.* Typhimurium-infected, anti-TNF-α treated, CT26 tumor-bearing BALB/c mice (grey bars). Error bars show standard deviations of means. (b) Photographs of CT26 tumors of an *S.* Typhimurium-infected tumor (left set of pictures) and an *S.* Typhimurium-infected, anti-TNF-α treated tumor (right set of pictures). The left column of each set of pictures show the fur side of the tumors, the right pictures show the ventral side of the tumors. (c) Hemoglobin content in CT26 tumors of *S.* Typhimurium-infected BALB/c mice (black bars) and *S.* Typhimurium-infected, anti-TNF-α treated BALB/c mice (grey bars). Error bars show standard deviations of means. (*) At 6 h p.i. the difference of hemoglobin content in the tumors is significant with p<0.05. (d) Bacterial number per g tumor tissue in *S.* Typhimurium-infected tumor-bearing BALB/c mice (black bars) and *S.* Typhimurium-infected, anti-TNF-α treated tumor-bearing BALB/c mice (grey bars). Error bars show standard deviations of means. (*) At 12 h p.i. the difference of bacterial numbers between differently treated tumors is significant with p<0.05. Results are representative for at least two independent experiments with 3–5 mice per group. The second experiment also included an isotype control where normal rat IgG was injected into the mice of the control group (data not shown).

We then wanted to test whether TNF-α is the sole cytokine responsible for the blood influx and bacterial colonization of solid tumors. Therefore, we neutralized TNF-α by injecting rat anti-mouseTNF-α i.v. 20 min before infection. As intended, the majority of TNF-α in blood was neutralized this way (Fig. 5a). The consequence was a retardation of blood influx into the tumor (Fig. 5b). While tumors from Salmonella-infected mice became dark red within 2 h p.i., tumors of anti-TNF-α treated mice began to show this phenomenon between 4 and 6 h p.i.. No resolution of blood influx became apparent within 30 h p.i..

These observations were in agreement with hemoglobin content in tumors of both groups of mice (Fig. 5c). While in Salmonella-infected mice the hemoglobin content in tumors quickly increased to a maximum between 6 h and 12 h p.i. and quickly decreased thereafter, the hemoglobin content in tumors of anti-TNF-α-treated, Salmonella-infected mice increased more slowly and did not decrease within the observation period.

Finally, the bacterial tumor-colonization from both groups was determined by plating tumor homogenates (Fig. 5d). While *Salmonellae* quickly accumulated and grew in CT26 tumors, tumor colonization after treatment with anti-TNF-α was slightly delayed. 12 h p.i. around 5×10^6^ bacteria/g tumor were found in tumors of Salmonella-infected mice, but only around 7×10^5^ bacteria/g tumor were found in infected mice that were treated with anti-TNF-α. At later time points, bacterial numbers in tumors of both groups became similar. Thus, a severe diminution of TNF-α can delay tumor colonization by *S.* Typhimurium but does not abolish it. These data indicate that either minimal amounts of TNF-α are sufficient to induce blood influx and support bacterial tumor colonization or that the absence of TNF-α can be compensated by other factors that are induced during a bacterial infection.

### Expression of multiple cytokines in serum shortly after i.v. administration of *S.* Typhimurium

**Figure 6:**
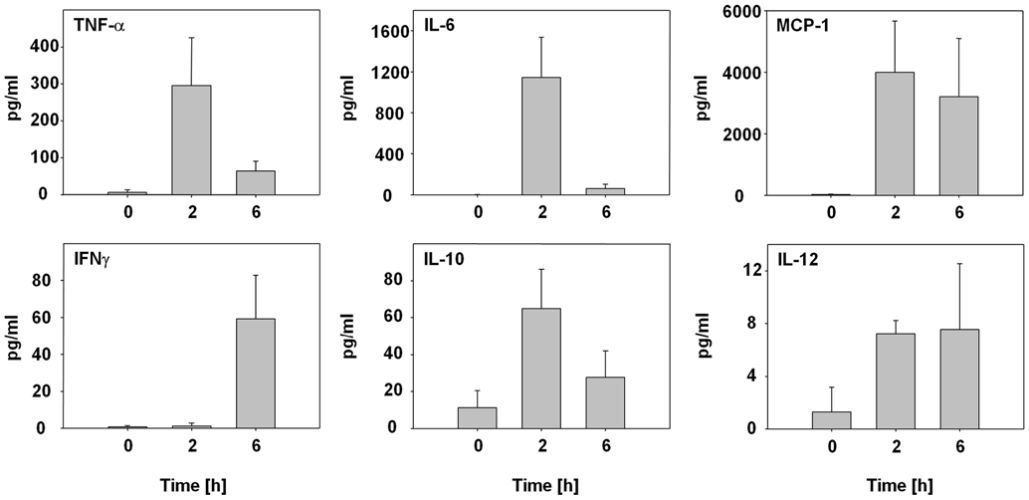
Measurement of different proinflammatory cytokines in the initial phase of bacterial tumor colonization. Concentrations of the proinflammatory cytokines TNF-α, IL-6, MCP-1, IFN-γ, IL-10 and IL-12(p70) in blood of uninfected (0 h) and *S.* Typhimurium-infected (2 h, 6 h) CT26 tumor-bearing BALB/c mice at different time points p.i.. Error bars show standard deviations. The differences between *S.* Typhimurium-infected mice and uninfected control mice are significant with p<0.01–0.05. The 6 h-values for IL-10 and IL-12 are not significantly different from non-infected mice. According to the manufacturer's protocol, the values for IL-12 can be considered background. The exact values for MCP-1 are higher than plotted here since several samples reached the plateau. Results are representative for two independent experiments with 3–5 mice.

Since depletion of TNF-α after i.v. administration of *S.* Typhimurium did not inhibit blood influx and bacterial tumor colonization completely, we suspected other pro-inflammatory cytokines to additionally support efficient bacterial tumor-colonization. Thus, we analyzed blood from infected and uninfected mice shortly after infection for selected cytokines (Fig. 6). As expected, several proinflammatory cytokines and chemokines such as IL-6 and MCP-1 are highly up-regulated by 2 h p.i.. Interestingly, the secretion pattern of IL-6 resembles the pattern of TNF-α, while MCP-1 and IL-12 plateaued later. A fourth mediator, IFNγ was up-regulated only 6 h p.i.. Interestingly, IL-10 an anti-inflammatory cytokine was also induced with an early rise.

Taken together, TNF-α is an important and effective factor in the initial phase of bacterial tumor-colonization, but it is probably not the only cytokine that is involved in bacterial entry into solid tumors.

## Discussion

*Salmonellae* have been tested systematically in experimental tumor therapies for more than 10 years now [3]. However, little is known about the mechanisms that are involved in bacterial tumor-colonization, i.e. how *Salmonellae* escape from circulation into the tumor and which factors are involved.

The present work investigates initial events of tumor-colonization by attenuated *S.* Typhimurium SL7207. Bacteria can be detected histologically in blood vessels of CT26 tumors already between 30 min to 2 h after systemic administration. In rare cases, they are found inside tumor tissue but still in close proximity to blood vessels.

Tumor blood vessels are structurally irregular and leaky, with irregular diameters and abnormal branching patterns [32], [33]. They exhibit a defective cellular lining composed of disorganized, loosely connected, branched or overlapping endothelial cells [34]. The openings between such disorganized cells do not only contribute to leakiness of blood vessels in tumors but also permit access of macromolecules to the tumor tissue. The size of these openings ranges between 200 and 2000 nm [34]. By extrapolation, it can be assumed that bacteria can also access tumors via such openings. Thus, escape of single bacteria from the blood stream into tumor tissue could be passive or active. In a passive escape scenario, bacteria would enter the tumor via the described openings by coincidence. In an active scenario they would actively seek the openings due to chemotaxis [26]. Also, a combination of both scenarios appears plausible.

Shortly after the escape of single bacteria into tumor tissue, invaded bacteria apparently proliferate. By 6 h p.i. micro-colonies can be found in the vicinity of blood vessels which become larger as time proceeds. By 15 h p.i. bacteria start to disseminate throughout the tumor. This initial phase is accompanied by a strong influx of blood into the tumor.

It was assumed that the bacterial infection induces the expression and secretion of cytokines in the tumor. A prime candidate was TNF-α, which is known to act as a vasoactive agent by modifying the actin cytoskeleton and increasing the permeability of the endothelial lining of blood vessels [31]. Contrary to our original expectation, TNF-α expression was neither detectable in infected tumor cells nor in colonized tumors. Instead, TNF-α was detectable between 30 min to 2 h p.i. at high concentrations in blood of infected tumor-bearing mice. The cells responsible for production of TNF-α could not be indentified thus far. According to the colonization data, the source of TNF-α could be infected cells of spleen or liver. Blood cells could also be responsible, although most blood-borne bacteria were not associated with cells (data not shown). In any case, TNF-α found in serum at 30 min is most likely derived from cells with pre-stored pools of this cytokine. Novel bio-synthesis should not be possible within such a short period of time but could account for TNF-α at the later time points.

TNF-α induced by intravenously administered *Salmonella* could apparently account for the initiation of blood influx into the tumors since we could show that TNF-α alone is sufficient for this reaction. However, depletion of TNF-α by antibodies had only a partial effect on blood influx and bacterial tumor-colonization. Our finding that several additional cytokines were detected in blood simultaneously with TNF-α might be the explanation. However, despite the presence of antibodies, residual free active TNF-α cannot entirely be excluded to be a possible reason for the remaining hemorrhage since extremely small amounts suffice to induce blood influx into tumors.

On the other hand, it is not surprising that intravenous application of Gram-negative bacteria activates a cascade of pro-inflammatory cytokines in the host that may account, in addition, for the induction of other effector molecules observed during bacterial colonization of tumors like MMP9 etc. [27]. For further improvement of bacterial tumor therapy it might be required to obtain a global picture of the events during the tumor invasion by applying genomics based approaches. In addition, it might be required to assess the role of every single cytokine and molecule induced under these conditions by employing appropriate knock out mice.

Nevertheless, our findings suggest that this initial burst of cytokines induced after systemic administration of *Salmonellae* is an essential reaction for efficient bacterial invasion of tumors. Thus, attenuation of *Salmonellae* might render the bacteria safe with respect to sepsis. On the other hand this might be achieved at the expense of invasion efficiency.

First clinical trials were carried out with the highly attenuated Salmonella strain VNP20009 that induces 10.000-fold less TNF-α in human peripheral blood cells [20]. The low efficiency of tumor-colonization observed in patients might be due to an over-attenuation that might not only affect the induction of TNF-α but the inflammatory response in general. This is an important point that urgently needs clarification.

Other attempts have been made to improve *Salmonella* by special attenuations. For example, a genetically-modified *Salmonella* Typhimurium strain that is auxotrophic for the amino acids arginine and leucine has been used in several studies showing strong tumor colonization while liver and spleen are cleared rapidly [4]. After reisolation from a tumor this auxotrophic strain showed colonization of not only necrotic but also viable tumor regions and was able to induce tumor regression or eradication in several tumor models [12], [35]–[37]. As this strain showed a great potential in the model systems used so far, it is of special interest to see its behavior in immunocompetent mice.

Another issue of this work addresses the establishment of necrotic regions and development of a viable tumor rim in solid tumors after bacterial colonization. Whenever analyzing bacteria-colonized tumors, bacteria were mainly restricted to necrotic regions, while an outer rim of viable tumor cells invariably persists [25], [28]. The large, bacteria-colonized necrosis is likely a result of the infection-induced hemorrhage in the tumor. Tissue in vicinity of hemorrhage becomes necrotic as can be seen in HE-stained paraffin sections between 9 h and 15 h p.i..

A second cause of the emerging necrosis could be the disintegration of blood vessels in the hemorrhagic region resulting in collapse of blood flow. As a result, the constant supply of tumor cells with nutrients and oxygen ceases. Thus, the tumor cells in the center of the tumor are “starved and suffocated to death”. In contrast, the viable rim consists of the tumor tissue that was not in contact with inflowing blood and therefore is still supplied with nutrients and oxygen.

Interestingly, the same phenomenon of formation of necrotic regions and a viable tumor rim was reported for solid tumors that were treated with VDAs [38]–[40]. Tumor center and periphery can exhibit different interstitial blood pressure and vascular architecture. Thus, tumor periphery might be less sensitive to vascular shutdown than the center. As the interstitial blood pressure rises strongly from tumor periphery to tumor center, an increase in vascular permeability might be catastrophic in the center, while it is tolerated in the periphery. In addition, blood vessel density at the tumor periphery is usually higher compared to the center. In case of extensive vascular damage, residual blood flow is likely to persist in the tumor periphery, but less likely in the tumor [39].

Having shown the involvement of TNF-α acting as a VDA in the initial phase of tumor colonization by *S.* Typhimurium, the similarity between VDAs and tumor-targeting bacteria in experimental tumor therapies becomes comprehensible. Although the increase in vessel permeability is induced differently in both therapies, the outcome is similar.

Taken together our results show that TNF-α among other pro-inflammatory cytokines plays an important role in the early phase of bacterial tumor-colonization. They further indicate that an initial modification of the observed “concert of proinflammatory cytokines” can adversely affect the efficiency of bacterial tumor colonization.
